# Minicircle DNA vector expressing interferon-lambda-3 inhibits hepatitis B virus replication and expression in hepatocyte-derived cell line

**DOI:** 10.1186/s12860-020-00250-9

**Published:** 2020-02-18

**Authors:** Xiaoyan Guo, Dianke Chen, Qingxian Cai, Zhanlian Huang, Wenxiong Xu, Liang Peng, Ping Chen

**Affiliations:** 1 Department of Infectious Diseases, The Third Affiliated Hospital of Sun Yat-Sen University, Guangzhou, China; 2 Department of Medical Oncology, The Sixth Affiliated Hospital, Sun Yat-Sen University, Guangzhou, China; 3 Department of Hepatology, The Third People’s Hospital of Shenzhen, Shenzhen, China; 4 Shenzhen Institutes of Advanced Technology, Chinese Academy of Sciences, Shenzhen, China

**Keywords:** Minicircle DNA, Type III interferon, Interferon-stimulated gene, Hepatitis B virus

## Abstract

**Background:**

Interferon-alpha (IFNα) is a first-line treatment option for chronic hepatitis B virus (HBV) infection, but the severe systemic side-effects limited its clinical application. Interferon-lambda (IFNλ) with comparable antiviral activity and less toxic side-effects is thought to be a good alternative interferon to IFNα. Additionally, the gene vector mediated sustainably expression of therapeutic product in the target cells/tissue may overcome the shortcomings resulted from the short half-life of IFNs.

**Results:**

We constructed a liver-specific IFNλ3-expressing minicircle (MC) vector under the control of a hepatocyte-specific ApoE promoter (MC.IFNλ3) and investigated its anti-HBV activity in a HBV-expressing hepatocyte-derived cell model (HepG2.2.15). As expected, the MC.IFNλ3 vector capable of expressing IFNλ3 in the recipient hepatocytes has demonstrated robust anti-HBV activity, in terms of suppressing viral antigen expression and viral DNA replication, via activation the interferon-stimulated gene (ISG) expression in HepG2.2.15 cells.

**Conclusions:**

Given the MC vector can be easily delivered into liver, the liver-targeted IFN gene-transfer (MC.IFNλ3), instead of systemic administrating IFN repeatedly, provides a promising concept for the treatment of chronic HBV infection.

## Background

Hepatitis B virus (HBV), the causative agent of hepatitis B, remains a major threat to public health. It’s estimated that more than 240 million people are chronically infected with HBV and over 780,000 people die annually from hepatitis B-related complications [1, 2]. To date, there are no cures for chronic hepatitis B (CHB), as the current treatments including the nucleos(t) ide analogues (NAs) and interferon-alpha (IFNα) therapy do not effectively clear HBV from the infected individuals [3]. The NAs targeting the HBV polymerase (or termed reverse transcriptase) can substantially inhibit HBV replication, but it fails to eliminate the pre-existing HBV persistence template—the covalently closed circular DNA (cccDNA) [4]. Apart from the ISG-associated inhibitory activity against HBV replication [5], it’s report that the IFNα at high concentration can degrade cccDNA in a noncytopathic manner [6, 7]. Thus, the IFNα therapy can occasionally result in functional cure of CHB in some patients, but it suffers severe systemic side-effects as well as poor response rate [4]. Collectively, it’s necessary to develop novel anti-HBV agents that can eliminate virus with minimal side-effects.

Since 2003, a new type of interferon that structurally resembles to cytokines IL-10 family members (namely type-III interferon or IFN-λ) has been identified and characterized, including IFNλ1 (or IL-29), IFNλ2 (or IL-28A) and IFNλ3 (or IL-28B) [8, 9]. Among the three human IFNλ isoforms, IFNλ3 was shown to have highest antiviral activity in hepatocyte cell model [10]. IFNλ and IFNα have distinct extracellular receptors but share similar intracellular Janus kinase/signal transducer and activation of transcription (JAK/STAT) signaling transduction pathway, in response to viral infection [11–13]. Unlike the ubiquitously expressed IFNα receptor; the IFNλ receptor primarily distributed on epithelial cells including hepatocytes while expressed little on hematopoietic cells, fibroblasts, microvascular endothelial cells, adipocytes and CNS cells [14]. With restricted target cell types, the application of IFNλ as antiviral agent is expected to has less side-effects than IFNα therapy, for example it is less likely to cause leukopenias that is common in IFNα therapy [12, 15, 16]. Recent clinical trials have demonstrated that the IFNλ therapy is effective and well-tolerable in human patients with chronic HBV/HDV or HCV infection [17–19]. A phase II clinical trial on patients with CHB illustrated that the pegylated IFNλ led to virological outcomes equivalent to pegylated IFNα while with a better tolerability [20, 21]. The phase II Lambda Interferon Monotherapy (LIMT) study sponsored by Eiger BioPharmaceuticals (NCT02765802) has evaluated the safety and efficacy of pegylated IFNλ administration for 48 weeks in chronic HDV patients. According to the interim results report, a significant (2-log) HDV-RNA decline was observed in majority of patients, while the adverse side-effects typically seen with INFα were fewer [19, 22]. These studies suggest that IFNλ may be a good alternative treatment against HBV infection.

Owing to the limited in vivo half-life, the IFNs (even for the PEGylated long-acting format) needs to be administrated repeatedly during the long course of treatment (several months), and consequently inconvenience their clinical application. The gene therapy that expressing IFNs in vivo by using a gene vector provides an alternative solution to bypass this limitation. As HBV is a liver tropic virus that specifically infect the hepatocytes, the chronic or persistent HBV infection can be viewed as an acquired genetic liver disease and it’s possible that CHB can be treated by a liver-targeted gene therapy [23]. In this study, we constructed a hepatocyte-specific minicircle DNA (MC) vector encoding IFNλ3 gene (MC.IFNλ3) and verified its anti-HBV activity in vitro. Where the MC [24] is an bacterial backbone DNA-free non-viral vector which permits stable and highly transgene expression in vitro and in vivo [25–28].

## Results

### MC.IFNλ3 permits hepatocyte-specific expression of IFNλ3

**Fig. 1:**
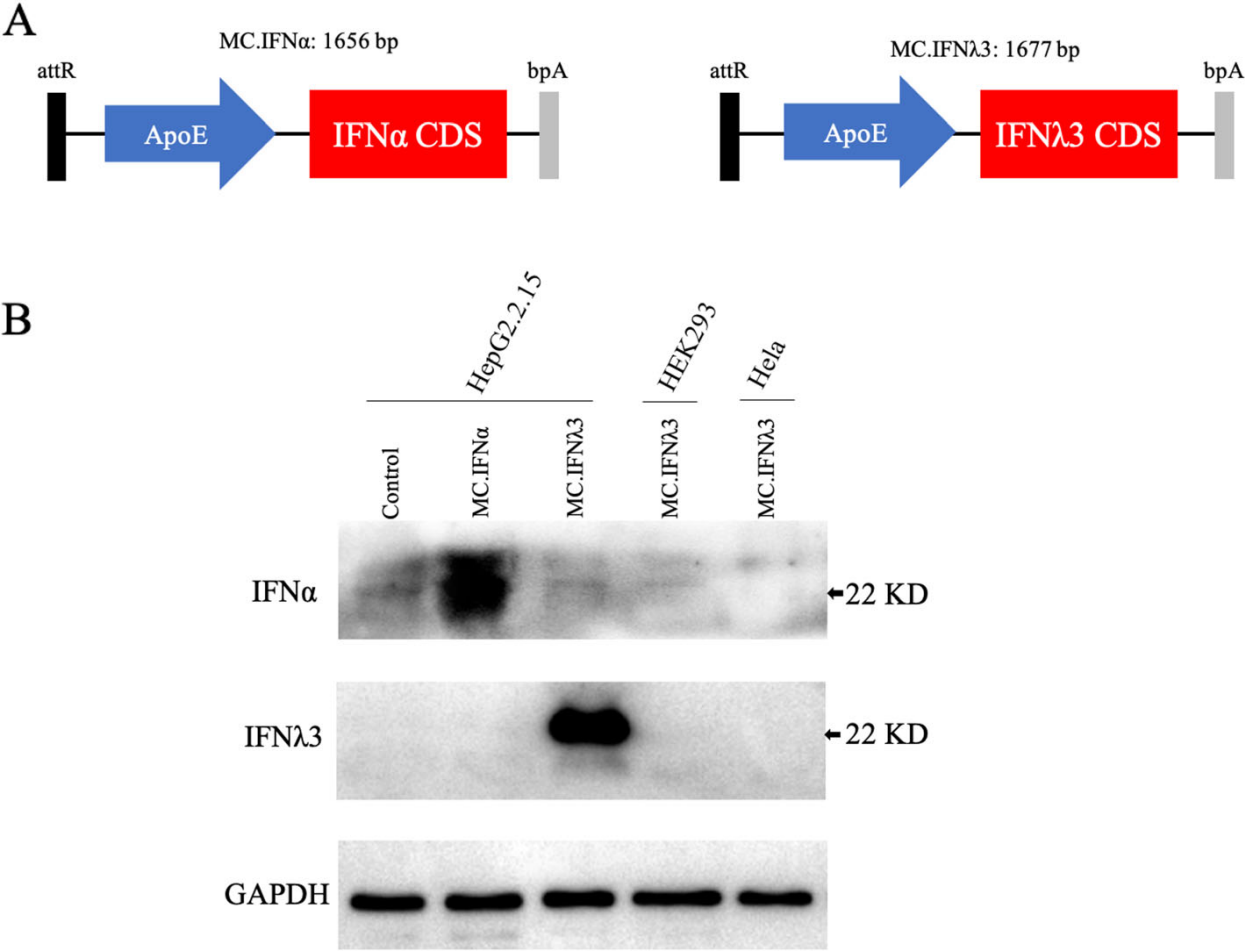
MC.IFNλ3 permits hepatocyte-specific expression of IFNλ3. HepG2.2.15, HEK293 and Hela cells were transfected with MC vectors. **a** Schematic illustration of the MC.IFNs. MC.IFNα is 1656-bp in length, MC.IFNλ3 is 1677-bp in length. attR represents a 36-bp attR recombinant site. ApoE indicates ApoE promoter. CDS represents coding sequence. bpA represents bovine growth hormone polyadenylation signal. **b** The expression of IFNα and IFNλ3 in cell lysate was determined by Western Blot at 3 days post-transfection. Lane 1–5 represents the untreated control (HepG2.2.15 cells without MC transfection), MC.IFNα transfected HepG2.2.15 cells, and MC.IFNλ3 transfected HepG2.2.15 cells, MC.IFNλ3 transfected HEK293 cells, MC.IFNλ3 transfected Hela cells, respectively

The MC.IFN⍺ (1656 bp in length; Fig. 1a left) or MC.IFNλ3 (1677 bp in length; Fig. 1a right) construct under the control of a ApoE promoter was designed to specifically express the corresponding interferon (IFN⍺ or IFNλ3) only in hepatocytes. To verify this assumption, we determined the expression of IFN⍺ or IFNλ3 in a variety of cell lines after 3 days of transfection with MC.IFNs by Western blot, including in HepG2.2.15 (hepatocyte), HEK293 (embryonic kidney cell) and Hela (Cervical squamous cell) cell lines.

Little or no IFN⍺/IFNλ3 signal was detected in MC transfected HEK293 or Hela cells while clear and strong protein signal was shown in the HepG2.2.15 cells transfected with MC.IFN⍺ (Fig. 1b upper row, Lane 2) or MC.IFNλ3 (Fig. 1b middle row, Lane 3), illustrating the MC.IFNs constructs permit hepatocyte-specific expression of interferons. The very weak signals of IFN⍺ presented in the untreated HepG2.2.15 cells (control) suggests that it may have baseline (low level) of endogenous IFN⍺ in the HepG2.2.15 cells (Fig. 1b upper row); in contrast, no baseline expression of endogenous IFNλ3 was detected in HepG2.2.15 cells (Fig. 1b middle row).

### MC.IFNλ3 inhibits viral antigens expression and viral DNA replication in HepG2.2.15 cells

To investigate the anti-HBV activity of the MC.IFNs, the viral DNA and secretory viral antigens (HBsAg and HBeAg) in cell culture supernatant from MC.IFNs transfected HepG2.2.15 cells were detected at 3- and 6 days after transfection. Where the transfection efficiency of HepG2.2.15 cells with MC.IFNs was roughly estimated to be about 70%, by using the MC vector, with comparable size (1.8 kb vs 1.7 kb), encoding an enhanced green fluorescent protein (MC.eGFP) as an indicator.

**Fig. 2:**
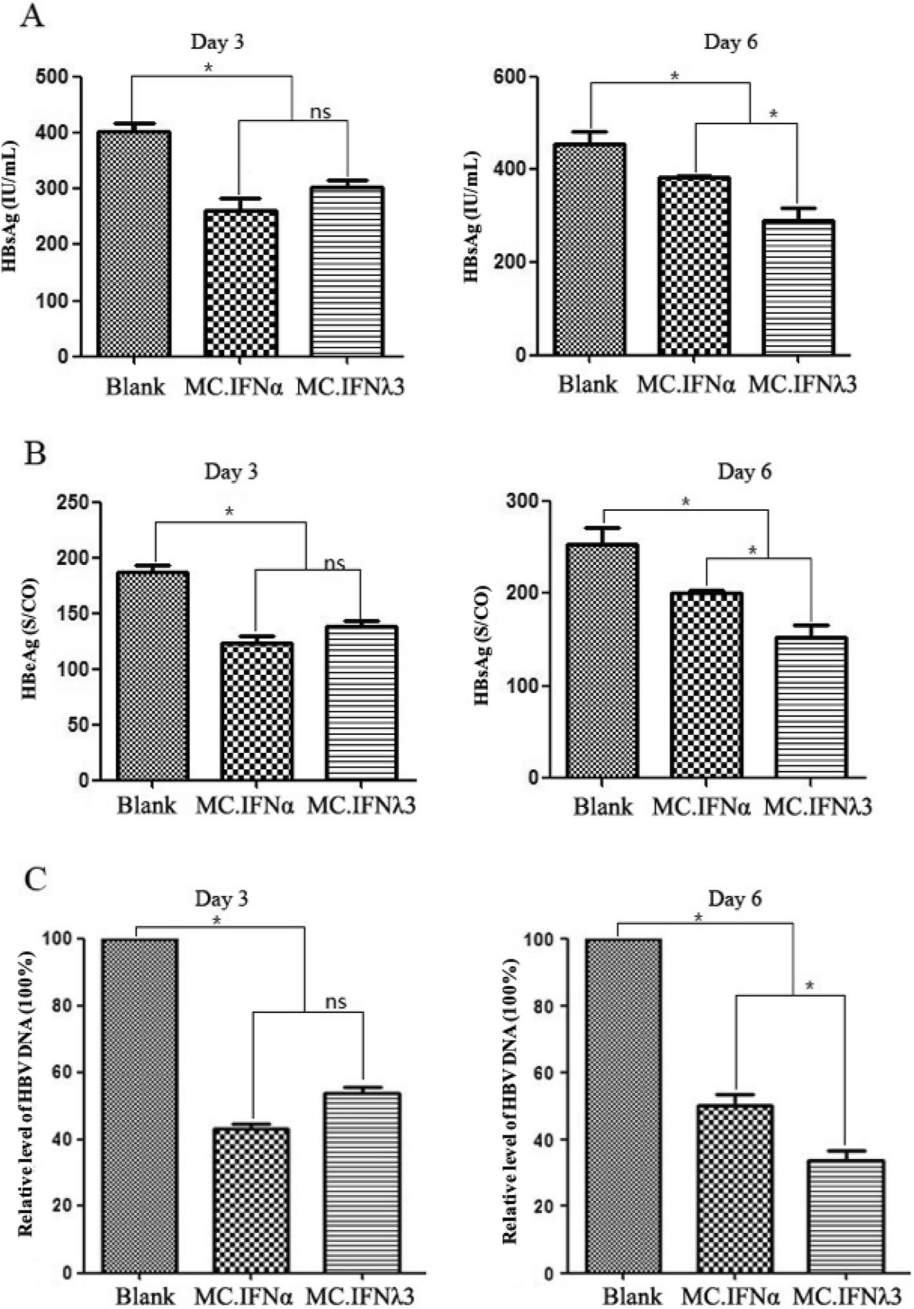
MC.IFNλ3 inhibits viral antigens expression and viral DNA replication in HepG2.2.15 cells. HepG2.2.15 cells were transfected with MC.IFNλ3 and MC.IFNα. While the untreated HepG2.2.15 cells served as a blank control (Blank). The levels of viral antigens, namely HBsAg (**a**) and HBeAg (**b**), and viral DNA in cell culture supernatant were determined by chemiluminiscence and qPCR, respectively, at the indicated time-points (3 or 6 days post-transfection). All data are shown as mean ± SD from three independent experiments. * indicates statistically significant (*P*-value < 0.05), ns indicates not significant (*P*-value > 0.05)

Like MC.IFN⍺, MC.IFNλ3 can inhibit both viral antigens (HBsAg and HBeAg) expression and viral DNA release (Fig. 2; Table 1). From a statistical perspective, MC.IFNλ3 and MC.IFN⍺ shows comparable anti-HBV activity at day 3 post-transfection (*P* > 0.05), although the inhibition rate of MC.IFNλ3 seems slight lower than that of MC.IFN⍺ (MC.IFNλ3 vs MC.IFN⍺ were 24.8% vs 35.1% for HBsAg, 26.5% vs 34.5% for HBeAg, 43.3% vs 53.6% for viral DNA); while after 6 days of transfection, MC.IFNλ3 shows statistically stronger (*P* < 0.05) anti-viral activities in comparison with its counterpart MC.IFN⍺, as the separate inhibition rates of viral antigens and viral DNA (MC.IFNλ3 vs MC.IFN⍺ were 36.7% vs 16.2% for HBsAg, 39.9% vs 20.9% for HBeAg, 50.3% vs 33.7% for viral DNA) (Table 1).

**Table 1:** Viral antigens and viral DNA in HepG2.2.15 cell culture supernatant after transfection

	Time Points	Group			*P*-value		
		Control (1)	MC.IFNα (2)	MC.IFNλ3 (3)	2 vs.1	3 vs.1	3 vs.2
HBsAg (IU/mL)	day 3	403.3 ± 26.2	261.4 ± 36.1	303.3 ± 20.6	**	**	ns
	day 6	456.4 ± 45.1	382.5 ± 5.8	288.9 ± 50.9	*	**	*
HBeAg (S/CO)	day 3	187.6 ± 10.7	122.8 ± 12.1	137.9 ± 9.4	**	**	ns
	day 6	253.4 ± 30.4	200.2 ± 5.3	152.2 ± 20.7	*	**	*
HBV DNA (100%)	day 3	1.00 ± 0.017	0.436 ± 0.034	0.536 ± 0.020	**	**	ns
	day 6	1.01 ± 0.005	0.50 ± 0.03	0.34 ± 0.03	**	**	*

** indicates *P* < 0.01; * indicates *P* < 0.05; ns represents not significant (*P* > 0.05). Group 1, 2 and 3 represent Control, MC.IFNα and MC.IFNλ3 group, respectively

### MC.IFNλ3 induces JAK1 and STAT1/STAT2 phosphorylation in HepG2.2.15 cells

**Fig. 3:**
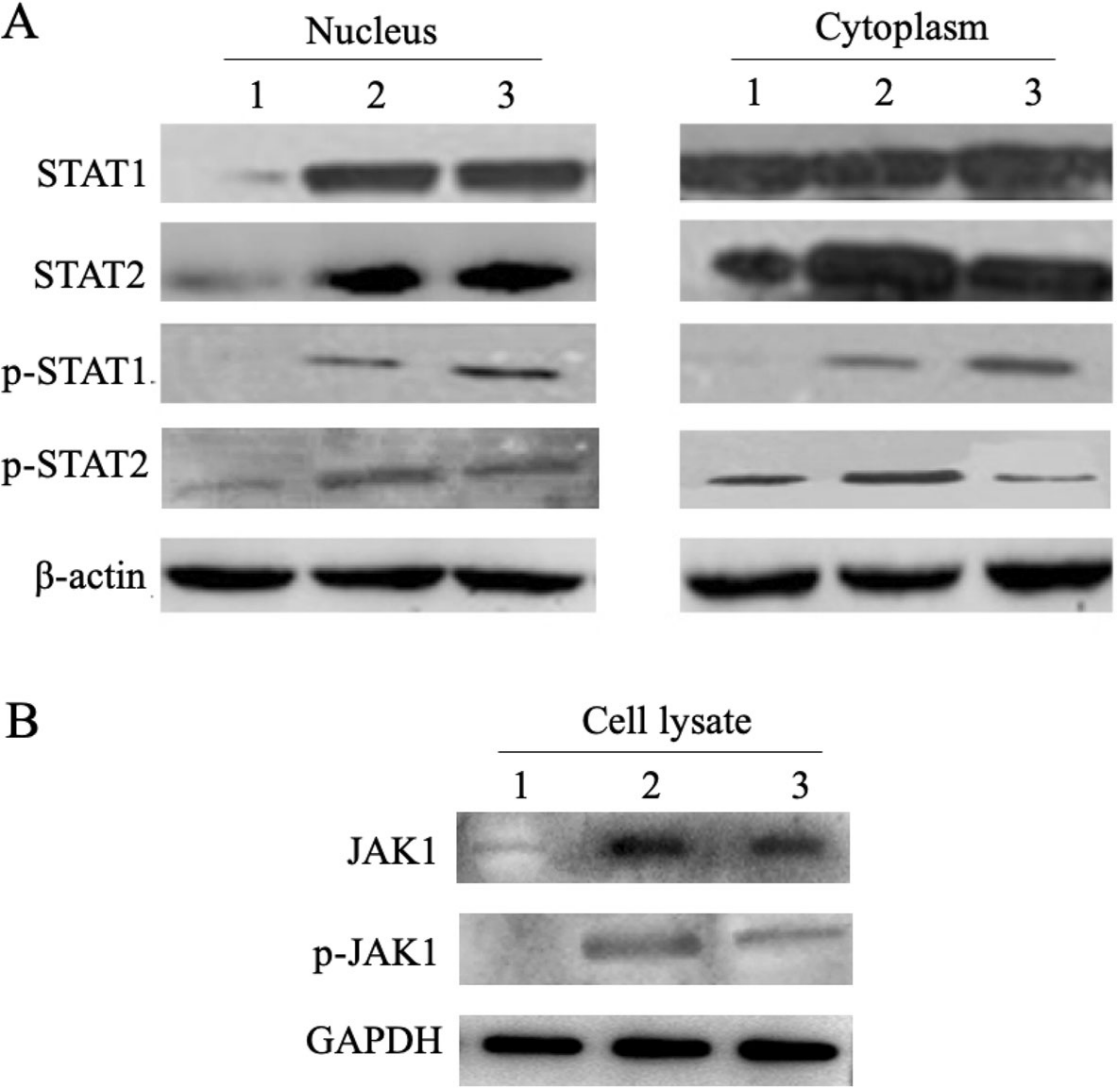
MC.IFNλ3 induce JAK1 and STAT1/STAT2 phosphorylation in HepG2.2.15 cells. HepG2.2.15 cells were transfected with MC vectors. The levels of **a** STAT1/STAT2 proteins and their phosphorylated form (p-STAT1/p-STAT2), **b** JAK1 and phosphorylated JAK1 (p-JAK1) in transfected HepG2.2.15 cells were determined by Western Blot at 6 days post-transfection. Lane 1, 2 and 3 represents untreated Control, MC.IFNα, and MC.IFNλ3 group, respectively

The un-phosphorylated and phosphorylated (p-STATs) form of STAT1/STAT2 both in cell nucleus and in cytoplasm of MC transfected HepG2.2.15 cells were determined by Western blot at 6 days post-transfection. The expression pattern differs significantly between cell nucleus (Fig. 3a left) and cytoplasm (Fig. 3a right). Except p-STAT1, STAT1, STAT2 and p-STAT2 are clearly expressed in the cytoplasm of the MC.IFNs-untreated cells (control) (Fig. 3a right). In contrast, the weak signals of STAT1, STAT2 and p-STAT2 in cell nucleus from the control samples also have been detected, indicating that there is baseline level of nuclear STAT1, STAT2 and p-STAT2 in the untreated cells (Fig. 3a left). For quantitative comparison of STATs/p-STATs among different groups, we estimated the relative levels of STATs/p-STATs by calculating the intensity of immunoblotting bands using the software Image J. We found that both MC.IFNs treatment dramatically increased the level of intra-nuclear STAT1 for about 13 (MC.IFN⍺) or 14 (MC.IFNλ3) times with a comparable level (MC.IFNλ3/MC.IFN⍺ = 1.06) (Fig. 3a). As comparable signals were detected among control and two MC.IFNs treated samples (control: MC.IFN⍺: MC.IFNλ3 = 0.9:1:1.2), we speculated that either MC.IFN⍺ or MC.IFNλ3 had little effect on the level of cytoplasmic STAT1 (Fig. 3a). The MC.IFNs treatment was also found to induce the comparably while significantly increase of the STAT2 levels both in cytoplasm (MC.IFN⍺ vs control: 2.9 times; MC.IFNλ3 vs control: 2.2 times; MC.IFN⍺/MC.IFNλ3 = 1.3) and nucleus (MC.IFN-⍺ vs control: 2.7 times; MC.IFNλ3 vs control: 3.1 times; MC.IFN⍺/MC.IFNλ3 = 1.1) for about 2 to 3 times (Fig. 3a). Given the cytoplasmic and nuclear p-STAT1 signals were presented in MC.IFN⍺ or MC.IFNλ3 treated cells but was absent in the control cells (Fig. 3a), it suggested that each MC.IFN can induce the phosphorylation of STAT1. Furthermore, the MC.IFNλ3 showed a stronger ability to activate phosphorylation of STAT1 (MC.IFNλ3/MC.IFN⍺ = 2.07 in cytoplasm; MC.IFNλ3/MC.IFN⍺ = 1.9 in nucleus) and both MC.IFNs were found to be able to comparably (MC.IFNλ3/MC.IFN⍺ = 1.02) elevate the nuclear p-STAT2 amount from baseline low level to a relative higher level for about 16 times (control: MC.IFN⍺: MC.IFNλ3 = 1:15.9:16.3) (Fig. 3a). These findings suggest that both MC.IFNs may up-regulate STAT2 expression, trigger the STAT1/STAT2 transferring from cytoplasm to nucleus and induce the phosphorylation of STAT1/STAT2.

To further investigate the activation of relevant upstream kinase of STAT1/STAT2 in JAK/STAT pathway, the JAK1 and phosphorylated JAK1 (p-JAK1) in MC transfected HepG2.2.15 cells were determined by Western blot at the same time point, namely 6 days post-transfection. Weak expression of JAK1 was shown in MC-untreated (control) cells (Fig. 3b upper row, Lane 1), while the increased expression of JAK1 in were observed in both MC.IFN⍺ and MC.IFNλ3 transfected cells (Fig. 3b upper row, Lane 2 and 3). On the other hand, the phosphorylated JAK1 (p-JAK1) was presented in both MC.IFNs treated cells (Fig. 3b middle row, Lane 2 and 3) but absent in the control cells (Fig. 3b middle row, Lane 1). These results suggest both MC.IFNs can up-regulate JAK1 expression and active the phosphorylation of JAK1.

Collectively, it’s clear that both MC.IFN⍺ and MC.IFNλ3 may activate JAK/STAT pathway in HepG2.2.15 cells.

### MC.IFNλ3 up-regulates ISGs expression in HepG2.2.15 cells

To further compare the ISGs expression profile alternation in HepG2.2.15 cells after MC.IFN treatment (MC.IFNλ3 vs MC.IFN⍺), the relative mRNA transcriptional levels of ten ISGs (IRF7, IRF9, Apobec3G, Mx1, BST2, PKR, OAS, IFT44, ISG15 and ISG56) of MC transfected HepG2.2.15 cells were quantified at 3 or 6 days post-transfection by qPCR.

**Fig. 4:**
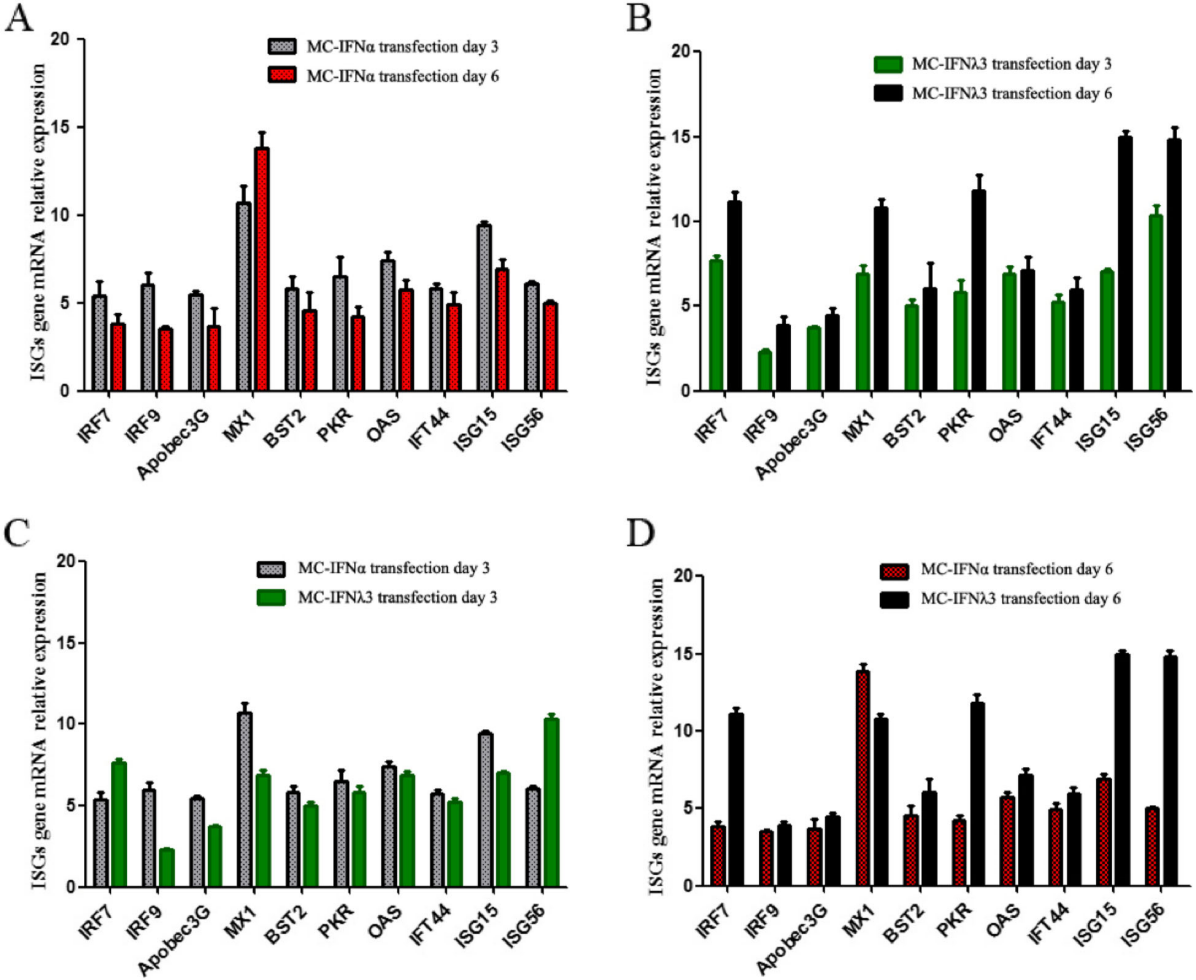
MC.IFNλ3 up-regulates ISGs expression in HepG2.2.15 cells. MC.IFNλ3 up-regulates ISGs expression in HepG2.2.15 cells. The relative mRNA transcriptional levels of ten ISGs MC transfected HepG2.2.15 cells were quantified at 3 or 6 days post-transfection by qPCR. The ISGs mRNA levels in HepG2.2.15 cells after MC.IFNλ3 (**a**) and MC.IFNα (**b**) treatment were compared between 3 days and 6 days post-transfection groups. The ISGs mRNA levels in HepG2.2.15 cells between MC.IFNλ3 and MC.IFNα treatment groups were compared at 3 days (**c**) or 6 days (**d**) post-transfection. All data are shown as mean ± SD from three independent experiments

Although with common feature that either MC up-regulated all the ten ISGs’ mRNA expression in each time-points (at 3 or 6 days post-transfection), the ISG expression profile under the induction of these two MC.IFNs showed significant different pattern across the time-course (Fig. 4). Firstly, we compared the change of mRNA relative expression level between two different time points (day 3 vs day 6 post-transfection). Compared with day 3, The expression of all but one (Mx1) ISGs, under MC.IFN-⍺ induction, at day 6 was decreased (Fig. 4a); while all the ISGs expression induced by MC.IFNλ3 is ever-increased over time (Fig. 4b). Furthermore, we compared the expression difference between two MC groups (MC.IFNλ3 vs MC.IFN⍺). In day 3, most ISGs (except IRF7 and ISG56) in MC.IFN⍺ groups expressed much more mRNAs than MC.IFNλ3 group (Fig. 4c); while it was completely reversed that the MC.IFNλ3 group expressed more mRNAs of all ISGs but Mx1 than MC.IFN⍺ group at day 6 post-transfection (Fig. 4d). These data demonstrated that, in comparison with IFN⍺, MC.IFNλ3 may induce a relative weaker ISGs-response in a short time, but the response is more robust in a prolonged period.

## Discussion

IFNλ has exerted significant antiviral activities against HBV or HCV [29–32] and is thought to be a potential alternative agent to IFNα against HBV/HCV infection [12]. Compared with IFNα that corresponds to ubiquitously expressed IFNα receptor, IFNλ may induce less side-effects as the IFNλ receptors are restrictedly expressed in epithelial cells including hepatocyte [14]. In fact, a recent clinical trial has showed that, compared to peg- to those of peg-IFNα, the PEGylated IFNλ exerts comparable serologic/virologic responses at end-of-treatment but less side-effects during on-treatment in CHB patients [20].

Given the long course of IFN-based anti-HBV therapies (months to 1 year), the IFNs with limited half-life are required to be repeatedly administrated weekly (pegylated) or more frequently [33, 34]; therefore, the clinical application of current IFNs is inconvenient and costly. Rather than extending the half-life, the gene therapy that persistently expressing IFN in vivo using an appropriate gene vector provides an alternative way to overcome these drawbacks. As HBV specifically infect the hepatocytes of the liver, chronic or persistent HBV infection can be considered as an acquired liver genetic disease. Thus, local gene expression of therapeutics product in the liver (or termed liver-targeted gene therapy) may be an attractive strategy against chronic HBV infection. By constructing a MC.IFNλ3 vector under the control of a liver-specific ApoE promoter that permits sustained IFNλ3 production in recipient hepatocyte cells, here we offered a liver-targeted long-acting alternative anti-HBV strategy. For liver-targeting, the non-viral MC vector, on one hand, can be delivered into liver easily via hydrodynamic tail vein injection [26, 35], the liver-specific ApoE promoter, on the other hand, will drive a specific expression of IFNλ3 in hepatocytes (Fig. 1).

In consistence with previous reports [11, 12], we confirmed that MC.IFNλ3, like the MC.IFNα counterpart, can induce efficient anti-HBV activity, in terms of suppressing HBV replication and expression, by activating the interferon-stimulated gene (ISG) expression (Fig. 4) through JAK/STAT pathway (Fig. 3). Furthermore, we found that, in comparison with MC.IFNα, MC.IFNλ3 induced a slightly weaker antiviral response in the earlier stage while a significant stronger antiviral response in the later stage, suggesting a robust inhibitory activity across the long course of IFNλ3 treatment (Fig. 2, Table 1).

We have noticed that the efficacy as well as the tolerance profiles of MC.IFNλ3 needs to be further evaluate in vivo with animal models. Nevertheless, our data are valuable for developing IFNλ3-based gene therapy against HBV infection.

## Conclusions

For chronic HBV infection treatment, the MC vector expressing IFNλ3 (MC.IFNλ3) provides a potential alternative strategy to the current IFN therapy.

## Methods

### Vector construction and minicircle DNA production

To construction the minicircle (MC) parental plasmid (PP) of IFNλ3 or IFNα, the coding sequences (CDS) of IFNλ3 and IFNα were separately sub-cloned into a modified minicircle-cloning vector pMC.BESXP [24] with additional hepatocyte-specific ApoE promoter, multiple cloning site (MCS) and bovine growth factor polyadenylation signal.

Using the standard MC preparation protocol described previously [24], the MCs encoding IFNλ3 (MC.IFNλ3) and IFNα (MC.IFNα) were produced in the *E. coli* strain ZYCY10P3S2T22 [24] transformed with corresponding parental plasmid.

### Cell culture and transfection

HEK293 cell, Hela cell and the HBV-positive HepG2.2.15 cell, purchased from Typical Culture Preservation Commission Cell Bank, Chinese Academy of Sciences (Shanghai, China), was maintained in Dulbecco’s modified Eagle’s medium (DMEM) supplemented with 10% fetal bovine serum (FBS) at 37 °C in a moist atmosphere containing 5% CO_2_. After 24 h of seeding at a density of 5 × 10^5^ cells per well of 6-well plates, the cells were transfected with 2 μg MC vector per well mixed with Lipofectamine 2000 (Invitrogen, US) according to the manufacturer’s instructions.

### Determination of viral DNA and antigens in cell culture supernatant

The level of secreted HBsAg and HBeAg in the cell culture supernatant was determined periodically by chemiluminiscence using the Abbott ARCHITECT platform (Abbott Laboratories, USA), according to the manufacturer’s instructions.

The HBV DNA in the cell culture supernatant was quantified by a TaqMax probe-based quantitative PCR method as performed according to the manufacturer’s instructions, using the COBAS® TaqMan® HBV Test Kit (Roche Diagnostics, US).

### Quantitative real-time PCR

The mRNA transcription level of ISGs was determined by quantitative real-time PCR. Total mRNA was isolated from the MC transfected cells at the indicated time points using TRIZOL (invitrogen, US). The RNA quantity and quality was measured using a NanoDrop2000 spectrophotometer (Thermo Scientific, US). Subsequently, cDNA was reverse transcribed and subjected to quantitative PCR (qPCR) with the SYBR® Premix Ex TaqTM II kit (TaKaRa, Japan). The ISG-specific qPCR primers are listed in Table 2.

**Table 2:** The qPCR primer pairs for detecting ISG genes

Genes	Primer pairs	
	Forward	Reverse
IRF9	gccctacaaggtgtatcagttg	tgctgtcgctttgatggtact
IRF7	gctggacgtgaccatcatgta	gggccgtataggaacgtgc
PKR	gccgctaaacttgcatatcttca	tcacacgtagtagcaaaagaacc
ISG56	ttgatgacgatgaaatgcctga	caggtcaccagactcctcac
IFNAR2	tcatggtgtatatcagcctcgt	agttggtacaatggagtggtttt
Mx1	gtttccgaagtggacatcgca	ctgcacaggttgttctcagc
OAS	ctgatgcaggaactgtatagcac	cacagcgtctagcacctctt
ISG15	cgcagatcacccagaagatcg	ttcgtcgcatttgtccacca
IFI44L	agccgtcagggatgtactataac	agggaatcatttggctctgtaga
IFITM1	ccaaggtccaccgtgattaac	atgaaccacattgtgcaaacct
Apobec3G	gcatcgtgaccaggagtatga	gtcagggtaaccttcgggt
β-actin	gccgggacctgactgacacctcat	tttgcggtggacgatggagg

The thermal cycling conditions were as follows: 30s at 95°С, followed by 40 cycles of 95°С for 10 s, 55°С for 10 s, and 72°С for 15 s. The relative abundance of a given transcript was estimated using the 2^-ΔΔ^Ct method, following normalization to ß-actin.

### Western blot

The protein samples were separated by SDS-PAGE and then transferred to a polyvinylidene difluoride (PVDF) membrane (Millipore, US). After blocked the non-specific binding sites with 5% skim milk in TBST (Sigma, US), the membrane was subjected to immunoblotting using a primary antibody listed as below: the rabbit polyclonal antibody specific to IFN⍺ (ProteinTech, US; #18013–1-AP) and IFNλ3 (ProteinTech, US; #24199–1-AP); the rabbit monoclonal antibodies specific to JAK1 (Cell Signaling Technology, US; #3344) and phosphorylated JAK1 (p-JAK1) (Cell Signaling Technology, US; #3331); the rabbit polyclonal or monoclonal antibodies specific to STAT1 (Abcam, UK; #ab2415), STAT2 (Abcam, UK; #ab53149), phosphorylated STAT1 (p-STAT1) (Cell Signaling Technology, US; #9171) and phosphorylated STAT2 (p-STAT2) (Millipore, US; #07–224). Finally, horseradish peroxidase-conjugated goat-anti-rabbit IgG secondary antibody (ProteinTech, US) and chemiluminescence system ECL Kit (Thermo Scientific, US) were used to visualize protein signal. For normalization, the housekeeping protein β-actin or GAPDH present on the same blots was detected using an anti-β actin antibody (ProteinTech, US) or anti-GAPDH antibody (Kangcheng BioTech, Shanghai, China).

The relative quantification of detected proteins on Western blotting was performed with the software Image J (https://imagej.nih.gov/ij/download.html) by estimating the intensity (or termed gray scale) of corresponding bands.

### Statistical methods

Mean and SD (or SEM) was calculated for each dataset. The statistical difference between two experimental groups (MC.IFN⍺ vs MC.IFNλ3) were compared using Student’s t-test; while the statistical comparison among multiple groups (≥ 3 groups) were performed with one-way ANOVA, following a Dunnett’s post-hoc tests. *P* value < 0.05 (*) was considered statistically significant. All these analyses were performed with Graphpad Prism 8 software (GraphPad Software, Inc., San Diego, CA).

## Data Availability

Not applicable.

## Abbreviations

cccDNA: covalently closed circular DNA
CDS: Coding sequences
hAAT: human alpha-1 antitrypsin
HBeAg: Hepatitis B e antigen
HBsAg: Hepatitis B surface antigen
HBV: Hepatitis B virus
HCV: Hepatitis C virus
HDV: Hepatitis D virus
IFNα: Interferon-alpha
IFN-λ: Interferon-lambda
ISG: Interferon-stimulated gene
JAK/STAT: Janus kinase/signal transducer and activation of transcription
MC: Minicircle
MCS: Multiple cloning site
NAs: Nucleos(t) ide analogues
PP: Parental plasmid
qPCR: quantitative Polymerase Chain Reaction
