# Correction to: Minicircle DNA vector expressing interferon-lambda-3 inhibits hepatitis B virus replication and expression in hepatocyte-derived cell line

**DOI:** 10.1186/s12860-020-00255-4

**Published:** 2020-03-02

**Authors:** Xiaoyan Guo, Dianke Chen, Qingxian Cai, Zhanlian Huang, Wenxiong Xu, Liang Peng, Ping Chen

**Affiliations:** 1 Department of Infectious Diseases, The Third Affiliated Hospital of Sun Yat-Sen University, Guangzhou, China; 2 Department of Medical Oncology, The Sixth Affiliated Hospital, Sun Yat-Sen University, Guangzhou, China; 3 Department of Hepatology, The Third People’s Hospital of Shenzhen, Shenzhen, China; 4 Shenzhen Institutes of Advanced Technology, Chinese Academy of Sciences, Shenzhen, China

**Correction to: BMC Mol Cell Biol**

**https://doi.org/10.1186/s12860-020-00250-9**

Following publication of the original article [1], the authors reported an error that occurred during the production process. The α (alpha) character was erroneously omitted in the PDF version of the article.

The original article [1] has been corrected.

## References

[1] Guo X, Chen D, Cai Q (2020). Minicircle DNA vector expressing interferon-lambda-3 inhibits hepatitis B virus replication and expression in hepatocyte-derived cell line. BMC Mol Cell Biol.

